# Chemotherapy improves survival for patients with lymph node-negative invasive papillary breast cancer with tumors ≥2 cm: a SEER population-based study

**DOI:** 10.1093/oncolo/oyaf422

**Published:** 2025-12-22

**Authors:** Yizi Zheng, Zhuozhao Zhan, Tingting Wu, Jiaqi Ying, Aina Zheng

**Affiliations:** Department of Breast Surgery, Zhejiang Key Laboratory of Intelligent Cancer Biomarker Discovery and Translation, The First Affiliated Hospital of Wenzhou Medical University, Wenzhou, Zhejiang 325000, China; Department of Thyroid and Breast Surgery, The First Affiliated Hospital of Shenzhen University, Shenzhen Second People’s Hospital, Shenzhen University, Shenzhen, Guangdong 518035, China; Department of Mathematics and Computer Science, Eindhoven University of Technology, Eindhoven, AZ 5612, The Netherlands; Department of Breast Surgery, Zhejiang Key Laboratory of Intelligent Cancer Biomarker Discovery and Translation, The First Affiliated Hospital of Wenzhou Medical University, Wenzhou, Zhejiang 325000, China; Department of Breast Surgery, Zhejiang Key Laboratory of Intelligent Cancer Biomarker Discovery and Translation, The First Affiliated Hospital of Wenzhou Medical University, Wenzhou, Zhejiang 325000, China; The Operating Theater, The First Affiliated Hospital of Wenzhou Medical University, Wenzhou, Zhejiang 325000, China

**Keywords:** breast cancer, invasive papillary carcinoma, chemotherapy, prognosis

## Abstract

**Background:**

Invasive papillary breast cancer (IPC) is a rare cancer known to have a better prognosis than other breast cancers. This study aimed to explore whether patients with early-stage IPC can benefit from chemotherapy.

**Materials and Methods:**

IPC cases diagnosed between 2003 and 2021 were retrieved from the Surveillance, Epidemiology, and End Results (SEER) database. The Kaplan–Meier method and the Cox proportional hazard model were performed to identify significant prognostic factors. To eliminate selection bias and baseline characteristics, propensity score matching (PSM) was used. The primary endpoints were overall survival (OS) and breast cancer-specific survival (BCSS).

**Results:**

A total of 1892 patients were enrolled, and 273 pairs were screened after PSM. Multivariable analysis showed that age under 50, married status, smaller tumor size, negative lymph node (LN) status, and receipt of surgery, chemotherapy or radiotherapy were independently associated with better OS. After PSM, patients receiving chemotherapy had significantly improved OS (*P *= .012), while BCSS showed no significant difference (*P *= .099). After stratified by tumor size and LN status, chemotherapy could significantly improve the OS of patients with LN-negative IPC with tumors ≥ 2.0 cm (*P *< .001). Among the patients with grade III or undifferentiated diseases, the OS of the chemotherapy group was significantly better than that of the non-chemotherapy group (*P *< .001).

**Conclusions:**

For patients with LN-negative IPC with tumor ≥ 2.0 cm and patients with grade III or undifferentiated disease, chemotherapy significantly improved OS. Future randomized controlled trials are expected to validate the results.

Implications for Practice Conducting prospective studies on IPC to assess the efficacy of chemotherapy remains challenging due to its rarity. Leveraging a large population-based dataset, our study demonstrated that chemotherapy significantly improved overall survival in patients with lymph node-negative IPC with tumors ≥ 2 cm, as well as those with grade III or undifferentiated disease. Chemotherapy should be considered in these populations to avoid undertreatment.

## Introduction

Invasive papillary carcinoma (IPC) of the breast represents a rare pathological subtype of breast cancer. According to the World Health Organization’s (WHO) classification of breast tumors, as delineated in the fifth edition published in 2019, IPC is characterized as “invasive adenocarcinoma with papillary structure constituting greater than 90% of the invasive components.”^1^ This subtype accounts for approximately 0.5% to 2% of all invasive breast cancers.^2–4^

Previous studies have found that IPC has higher rates of estrogen receptor (ER) and progesterone receptor (PR) positivity, decreased rates of human epidermal growth factor receptor 2 (HER2) amplification, and a lower mean Ki-67 proliferation index,^5^^,^^6^ which might indicate a better prognosis.^7^ Endocrine therapy may be sufficient for a proportion of IPC patients with positive expression of hormone receptors.^8^ However, some patients still need to consider targeted therapy or chemotherapy.^9^ Some IPCs could be potentially invasive and show an increased risk of local recurrence and distant metastasis, especially those diseases with larger tumor size, involved lymph nodes, and positive HER2 expression.^10^ The benefits of chemotherapy for patients with lymph node-negative, small-sized invasive papillary breast cancer are still unclear. A previous study conducted by our team found that the advantage of favorable prognoses in IPC compared to those observed in the overall infiltrating ductal carcinoma (IDC) population was diminished after adjusting for demographic and clinicopathological factors.^11^ Therefore, patients diagnosed with IPC should be made aware that its biological features are not as favorable as once thought, and IPC patients might not be completely exempted from chemotherapy.

Currently, there is no established and unified optimal therapeutic strategy for IPC due to insufficient evidence-based medical research. Due to the rarity of invasive papillary carcinoma (IPC), most existing studies are case reports or small retrospective series, which provide limited evidence to guide the clinical management of IPC.^5^^,^^12^ However, most studies included patients diagnosed with IPC before 2003, which differs from the WHO’s current definition of IPC. Therefore, due to the influence of misclassification bias, more research is needed to verify whether the study results can still provide clinical reference. The rarity of the disease and its specious prognostic feature have resulted in a reduction of limited data from studies related to IPC, which has made clinical management more challenging to balance the risks of overtreatment versus undertreatment. At present, the existing treatment scheme for IPC is mainly based on IDC,^5^ which does not take into account the specific biological behavior of IPC. A larger sample study is awaited in advance to explore the indication of chemotherapy for IPC, particularly in patients with lymph-node negative and small-sized disease.

This study obtained data from the Surveillance, Epidemiology, and End Results (SEER) database, a large cancer registry based on the American population, to analyze and determine the impact of chemotherapy on the prognosis of patients with early-stage IPC. Our objective was to establish the clinical foundations for recommending chemotherapy to particular populations.

## Materials and methods

### Database

Data was obtained from the SEER 18 registry, a cancer database that captures approximately 30% of the U.S. population. All data was publicly available, de-identified, and exempted from the review of the Institutional Review Board. The data was obtained through the latest SEER*Stat 8.4.3 software.

### Patients and variables

**Figure 1.**
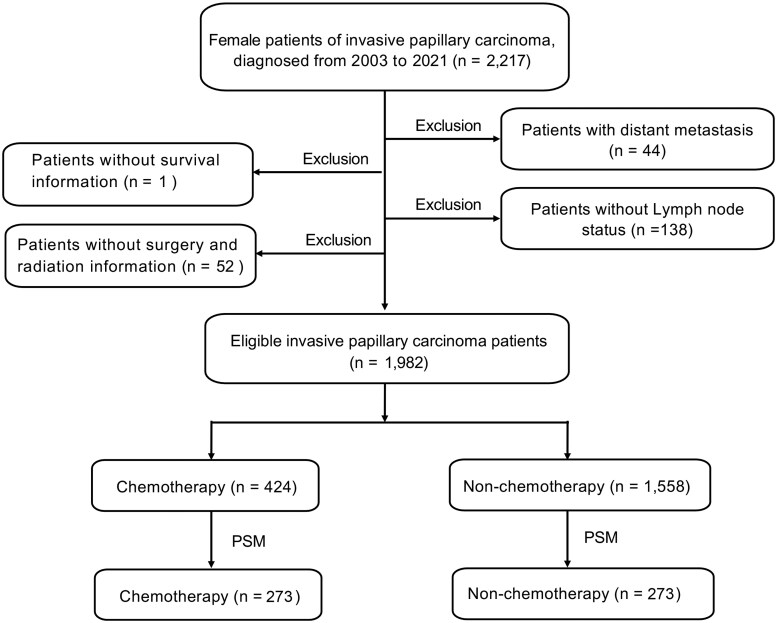
Flow chart for patient screening. SEER, Surveillance Epidemiology, and End Results; PSM, propensity score matching.

In this study, the criteria for inclusion of patients are as follows: female, pathologically diagnosed with IPC, unilateral breast cancer, one primary site only, diagnosed between 2003 and 2021. The pathologic diagnosis was based on the primary site according to the International Classification of Disease for Oncology, Third Edition (ICD-O-3). Patients with metastatic disease, missing survival data, and missing lymph node (LN) status were excluded. Between 2003 and 2021, there were 2217 female patients diagnosed with IPC. Among them, 44 patients with metastatic disease, 1 patient with missing survival information, 138 patients with missing lymph node status, and 52 patients with missing surgery and radiation information were excluded. This process resulted in 1982 eligible patients. The selection process is illustrated in Figure 1.

The causes of death were categorized as either breast cancer-related or non-breast cancer-related. The primary endpoints of the study were overall survival (OS) and breast cancer-specific survival (BCSS). OS was calculated from the date of diagnosis to the date of death caused by any reason or last follow-up. BCSS was defined as the time interval from the date of the diagnosis of breast cancer to the date of death caused by breast cancer. Before implementing any research work, approval from the Ethical Committee Review Board of the First Affiliated Hospital of Wenzhou Medical University had been obtained (No. KY2024-R269). The methods were performed in accordance with the principles stated in the Declaration of Helsinki. The data released by the SEER database do not require informed patient consent.

### Statistical analysis

Patients were divided into two groups according to whether they received chemotherapy or not (chemotherapy, *n* = 424; non-chemotherapy, *n* = 1558). The Pearson’s chi-squared test and Fisher’s test were used to analyze the clinicopathologic characteristics of patients in the chemotherapy group and the non-chemotherapy group. Independent prognostic factors related to OS and BCSS were determined by univariate and multivariate Cox proportional hazard regression analysis, and the corresponding hazard ratio (HR) and 95% confidence interval (CI) were reported. Propensity score matching (PSM) was employed to reduce potential selection bias and confounding in this observational study by balancing the baseline characteristics between the chemotherapy and non-chemotherapy groups. And the matching quality was tested. Fourteen predetermined factors were used for the propensity model to generate a matching ratio of 1:1 with 0.05 caliper width, including age, marital status, race, grade, laterality, stage, tumor size, LN status, breast subtype, ER status, PR status, HER2 status, surgery, and radiation. We consider it reliable to estimate the balance between the variables before and after PSM when the standardized difference is <10% or the *P*-value > .05. The Kaplan-Meier method with the log-rank test was used for survival analysis between the chemotherapy and non-chemotherapy groups. All data analysis was performed using R4.3.3 (Vienna, Austria; http://www.R-project.org). Two-sided *P*-values < .05 were considered to be statistically significant.

## Results

### Baseline patient characteristics

**Figure 2.**
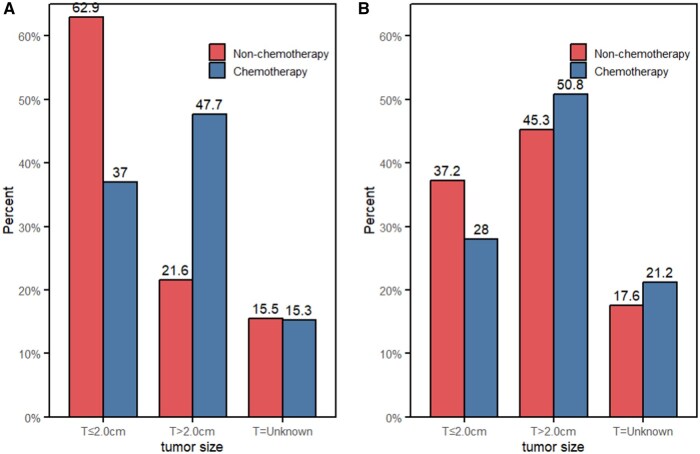
The distributions of the chemotherapy and the non-chemotherapy groups based on tumor size and lymph node status. (A) lymph node-negative group; (B) lymph node-positive group.

A total of 1982 female patients with breast IPC were eligible, of which 424 patients received chemotherapy and 1558 patients did not. The median follow-up time was 74 months (range: 1-263 months). The median age of patients was 66 years old, ranging from 23 to 90 years old, and 87.8% of patients were over 50 years old. Unmarried and married patients accounted for 46.7% and 48.1% of all patients, respectively (*P *< .001), and white patients accounted for 69.7% of all patients. In terms of molecular subtypes, 87.9% of patients were ER-positive and 81.4% were PR-positive (*P *< .001). Among 1155 cases with complete ER, PR, and HER2 data, 963 cases (83.5%) were hormone receptor-positive/human epidermal growth factor receptor 2-negative (HoR+/HER2−). Of all the patients, 67.2% were found to have grade I/II disease, 18.7% had grade III/IV disease, and the remaining 14.1% had disease of unknown grade. And 337 (17.0%) patients had positive LNs, while the other 1645 (83.0%) patients had negative LNs. Among the patients with negative LNs, only 14.3% received chemotherapy; among the patients with positive LNs, 56.1% received chemotherapy. Patients with negative LNs and tumors smaller than 2.0 cm were more common in the non-chemotherapy group (62.9% vs. 37.0%, *P *< .001, Figure 2A), while those with tumors larger than 2.0 cm were more common in the chemotherapy group (47.7% vs. 21.6%, *P *< .001, Figure 2A). When the patients had positive LNs, the proportion of patients with tumors smaller than 2.0 cm was similar in the chemotherapy and non-chemotherapy groups (37.2% vs. 28.0%, *P *= .112, Figure 2B), as was the proportion of patients with tumors equal to or larger than 2.0 cm (50.8% vs. 45.3%, *P *= .114, Figure 2B). More detailed clinical and pathological features of patients with IPC before and after PSM are shown in Table 1.

**Table 1.** Patients’ demographics and clinicopathological characteristics.

Variables	Data before PSM			Data after PSM		
	Non-chemotherapy (*n* = 1558)	Chemotherapy (*n* = 424)	*P*-value	Non-chemotherapy (*n* = 287)	Chemotherapy (*n* = 287)	*P*-value
**Age (%)**			<.001			.676
**<50**	119 (7.6%)	122 (28.8%)		60 (22.0%)	56 (20.5%)	
**≥50**	1439 (92.4%)	302 (71.2%)		213 (78.0%)	217 (79.5%)	
**Marital status (%)**			<.001			.568
**Married**	765 (49.1%)	161 (38.0%)		122 (44.7%)	111 (40.7%)	
**Unmarried^a^**	707 (45.4%)	246 (58.0%)		141 (51.6%)	149 (54.6%)	
**Unknown**	86 (5.5%)	17 (4.0%)		10 (3.7%)	13 (4.8%)	
**Race (%)**			.017			.988
**White**	1101 (70.7%)	280 (66.0%)		190 (69.6%)	190 (69.6%)	
**Black**	234 (15.0%)	90 (21.2%)		47 (17.2%)	45 (16.5%)	
**Other^b^**	202 (13.0%)	51 (12.0%)		34 (12.5%)	35 (12.8%)	
**Unknown**	21 (1.3%)	3 (0.7%)		2 (0.7%)	3 (1.1%)	
**Grade (%)**			<.001			.961
**I/II**	1145 (73.5%)	186 (43.9%)		155 (56.8%)	152 (55.7%)	
**III/IV**	170 (10.9%)	200 (47.2%)		88 (32.2%)	91 (33.3%)	
**Unknown**	243 (15.6%)	38 (9.0%)		30 (11.0%)	30 (11.0%)	
**Laterality (%)**			.507			.864
**Left**	811 (52.1%)	213 (50.2%)		139 (50.9%)	137 (50.2%)	
**Right**	747 (47.9%)	211 (49.8%)		134 (49.1%)	136 (49.8%)	
**Stage (%)**			<.001			.952
**I**	789 (50.6%)	84 (19.8%)		62 (22.7%)	64 (23.4%)	
**II**	190 (12.2%)	120 (28.3%)		71 (26.0%)	66 (24.2%)	
**III**	22 (1.4%)	47 (11.1%)		15 (5.5%)	17 (6.2%)	
**Unknown**	557 (35.8%)	173 (40.8%)		125 (45.8%)	126 (46.2%)	
**Tumor size (cm, %)**			<.001			.960
**≤2.0**	942 (60.5%)	140 (33.0%)		105 (38.5%)	108 (39.6%)	
**>2.0**	371 (23.8%)	208 (49.1%)		113 (41.4%)	110 (40.3%)	
**Unknown**	245 (15.7%)	76 (17.9%)		55 (20.1%)	55 (20.1%)	
**LN status (%)**			<.001			.482
**Negative**	1410 (90.5%)	235 (55.4%)		172 (63.0%)	164 (60.1%)	
**Positive**	148 (9.5%)	189 (44.6%)		101 (37.0%)	109 (39.9%)	
**ER status (%)**			<.001			.367
**Negative**	96 (6.2%)	143 (33.7%)		70 (25.6%)	61 (22.3%)	
**Positive**	1462 (93.8%)	281 (66.3%)		203 (74.4%)	212 (77.7%)	
**PR status (%)**			<.001			.284
**Negative**	173 (11.1%)	196 (46.2%)		104 (38.1%)	92 (33.7%)	
**Positive**	1385 (88.9%)	228 (53.8%)		169 (61.9%)	181 (66.3%)	
**HER2 status (%)**			<.001			.911
**Negative**	877 (56.3%)	192 (45.3%)		130 (47.6%)	125 (45.8%)	
**Positive**	30 (1.9%)	56 (13.2%)		19 (7.0%)	20 (7.3%)	
**Unknown**	651 (41.8%)	176 (41.5%)		124 (45.4%)	128 (46.9%)	
**Surgery (%)**			.001			>.999
**None**	77 (4.9%)	6 (1.4%)		6 (2.2%)	6 (2.2%)	
**Received**	1481 (95.1%)	418 (98.6%)		267 (97.8%)	267 (97.8%)	
**Radiotherapy (%)**			<.001			.392
**None**	847 (54.4%)	171 (40.3%)		138 (50.5%)	128 (46.9%)	
**Received**	711 (45.6%)	253 (59.7%)		135 (49.5%)	145 (53.1%)	

Abbreviations: ER, estrogen receptor; HoR, hormone receptor; HER2, human epidermal growth factor receptor 2; LN, lymph nodes; PR, progesterone receptor.

a Unmarried includes divorced, separated, single (never married), and widowed.

b Other include American Indian/Alaskan native or Asian/Pacific Islander.

### Survival analysis for OS and BCSS

**Figure 3.**
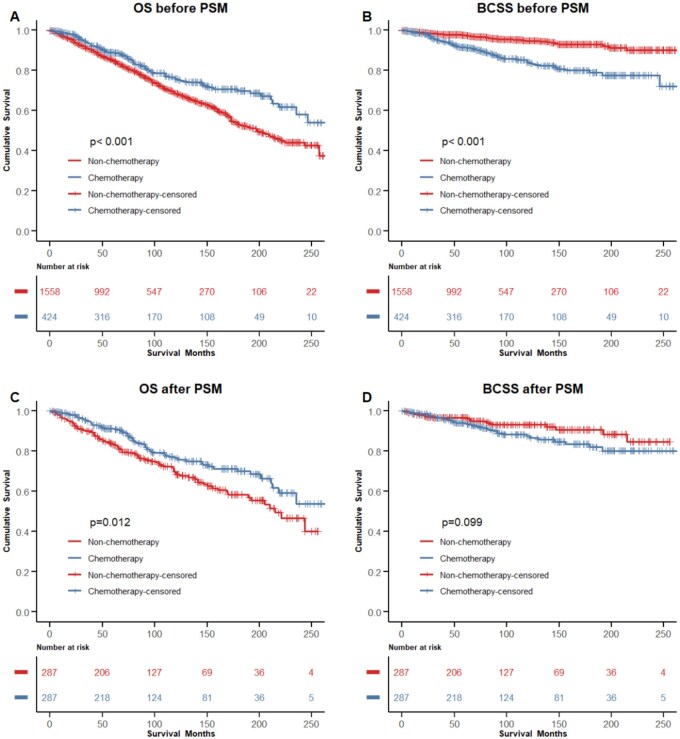
Comparison of OS and BCSS between the chemotherapy and the non-chemotherapy group. (A) OS before PSM; (B) BCSS before PSM. (C) OS after PSM; (D) BCSS after PSM.OS, overall survival; BCSS, breast cancer-specific survival; PSM, propensity score matching; T, tumor size; LN, lymph node.

Before PSM, OS in the chemotherapy group was significantly better than that in the non-chemotherapy group, while BCSS was significantly worse than that in chemotherapy group (OS, *P *< .001; BCSS, *P *< .001, Figures 3A and 3B). After eliminating the possible influence of confounding variables on OS and BCSS by PSM, there were 273 pairs of matched patients in the chemotherapy group and in the non-chemotherapy group. No demographic variables with significant differences were observed (*P *> .05). The OS in the chemotherapy group was still significantly better than that in the non-chemotherapy group (*P *= .012, Figure 3C), but there was no significant difference in BCSS between the two groups (*P *= .099, Figure 3D). The 5-year OS and BCSS of the two groups of patients before and after PSM were reported in **Table S1**. The 10-year OS and BCSS of the two groups are listed in **Table S2**. After PSM analysis, OS estimates were significantly better in the chemotherapy group than in the non-chemotherapy group (for 5-year OS, 91.3% vs. 83.4%, *P *= .0123; for 10-year OS, 76.6% vs. 68.5%, *P *= .0123).

**Figure 4.**
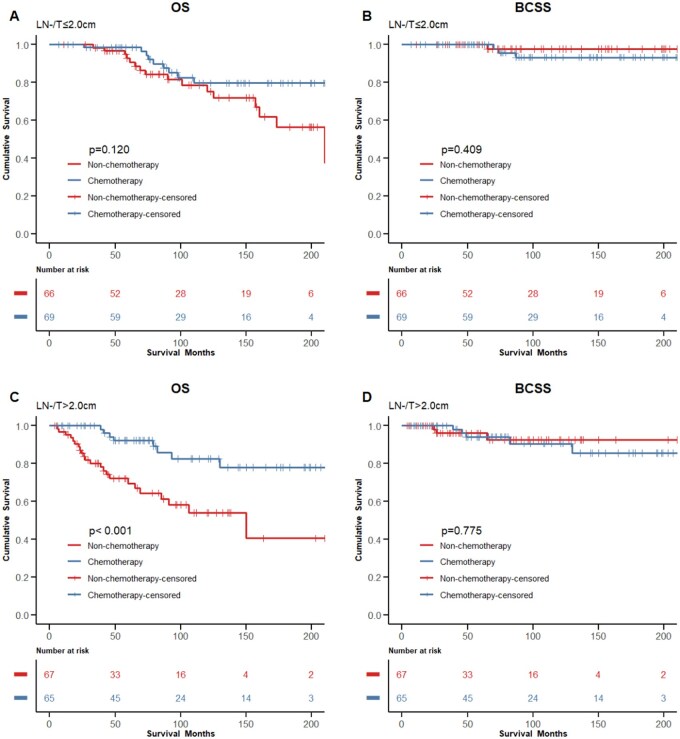
Comparison of the OS and the BCSS of LN-negative patients between the chemotherapy and the non-chemotherapy groups stratified by tumor size after matching. (A) OS in the T < 2.0 cm group; (B) BCSS in the T < 2.0 cm group; C, OS in the T ≥ 2.0 cm group; (D) BCSS in the T ≥ 2.0 cm group. OS, overall survival; BCSS, breast cancer-specific survival; T, tumor size; LN, lymph node.

Patients were then stratified into subgroups according to LN status and tumor size. Among the patients with negative LNs, the prognosis of the chemotherapy group was similar to that of the non-chemotherapy group when the tumor size ≤  2.0 cm (OS, HR = 0.527, 95%CI, 0.232-1.198, *P *= .120; BCSS, HR = 2.511, 95%CI, 0.261-24.150, *P *= .409, respectively; Figures 4A and 4B); whereas in patients with tumors ≥ 2.0 cm, adjuvant chemotherapy significantly improved OS but not BCSS (OS, HR = 0.265, 95%CI, 0.118-0.595, *P *< .001; BCSS, HR = 1.233, 95%CI, 0.293-5.191, *P *= .775, respectively; ­Figures 4C and 4D). For patients with positive LNs, chemotherapy showed no significant effects on OS and BCSS in patients with tumors smaller than 2.0 cm (OS, HR = 0.580, 95%CI, 0.209-1.607, *P *= .289; BCSS, HR = 1.392, 95%CI, 0.311-6.222, *P *= .664, respectively; Figures S1A and S1B); whereas in patients with tumors equal to or larger than 2.0 cm, OS in the chemotherapy group was seemingly better than the non-chemotherapy group but without statistical significance (OS, HR = 0.539, 95%CI, 0.258-1.126, *P *= .094; BCSS, HR = 0.670, 95%CI, 0.255-1.765, *P *= .416, respectively; ­Figures S1C and S1D).

Since histological grade reflects the malignancy of the tumor, patients were stratified into subgroups according to grade. In patients with grade I/II tumors, there was no significant difference in OS and BCSS between the chemotherapy and the non-chemotherapy groups (OS, HR = 1, 95%CI, 0.625-1.600, *P *= .999; BCSS, HR = 2.142, 95%CI, 0.872-5.262, *P *= .089, respectively; Figures S2A and S2B). Among the patients with grade III or undifferentiated diseases, the OS of the chemotherapy group was significantly better than that of the non-chemotherapy group, but not for BCSS (OS, HR = 0.370, 95%CI, 0.219-0.625, *P *< .001; BCSS, HR = 1.165, 95%CI, 0.535-2.539, *P *= .700, respectively; Figures S2C and S2D).

### Prognostic factors for invasive papillary carcinoma of the breast

In order to determine the prognostic factors for IPC of the breast, we conducted univariate and multivariate Cox analysis on the whole study cohort. Univariate analysis revealed that younger age, married status, other races, tumor size smaller than 2.0 cm, negative LN, ER-positive, PR-positive, surgery, radiation, and chemotherapy were all associated with improved OS (Table 2). The multivariate analysis revealed that the following were independent prognostic factors for OS (Table 2). In terms of basic information of the patients, age (age ≥ 50: HR = 2.677, 95% CI 1.868-3.837, *P *< .001), marital status (married, HR = 0.502, 95% CI 0.412-0.611, *P *< .001), and race (other: HR = 0.450, 95% CI 0.307-0.658, *P *< .001) were associated with OS. In terms of tumor-related information, tumor size (Tumor size > 2.0 cm: HR = 1.699, 95% CI 1.356-2.129, *P *< .001), and LN status (positive: HR = 1.496, 95%CI 1.189-1.884, *P *= .001) were related to OS. In terms of treatment information, surgery (received: HR = 0.352, 95% CI 0.249-0.499, *P *< .001), radiotherapy (received: HR = 0.514, 95% CI 0.421-0.627, *P *< .001), and chemotherapy (received: HR = 0.604, 95% CI 0.461-0.793, *P *< .001) were linked to OS.

**Table 2.** Univariate and multivariate Cox regression model analysis of OS between the chemotherapy group and the non-chemotherapy group.

Characteristics	Univariate analysis			Multivariate analysis		
	HR	95% CI	*P*-value	HR	95% CI	*P*-value
**Age**						
<50	Reference			Reference		
≥50	2.771	1.953-3.931	<.001	2.677	1.868-3.837	<.001
**Marital Status**						
Unmarried^a^	Reference			Reference		
Married	0.418	0.345-0.505	<.001	0.502	0.412-0.611	<.001
Unknown	0.724	0.481-1.089	.120	0.739	0.488-1.118	.153
**Race**						
White	Reference			Reference		
Black	1.207	0.972-1.500	.089	1.016	0.811-1.274	.888
Other^b^	0.440	0.301-0.642	<.001	0.450	0.307-0.658	<.001
Unknown	0.290	0.041-2.062	.216	0.171	0.024-1.225	.079
						.081
**Grade**						
I/II	Reference			Reference		
III/IV	1.177	0.946-1.464	.143	1.064	0.815-1.389	.650
Unknown	1.138	0.892-1.452	.297	0.923	0.715-1.191	.537
**Laterality**						
Left	Reference			Reference		
Right	1.003	0.841-1.196	.974	1.066	0.892-1.273	.484
**Tumor size**						
≤2.0	Reference			Reference		
>2.0	1.978	1.602, 2.442	<.001	1.699	1.356-2.129	<.001
Unknown	1.497	1.193, 1.879	<.001	1.431	1.123-1.824	.004
**LN status**						
Negative	Reference			Reference		
Positive	1.256	1.014-1.555	.037	1.496	1.189-1.884	.001
**ER status**						
Negative	Reference			Reference		
Positive	0.763	0.600-0.969	.026	0.810	0.556-1.178	.270
**PR status**						
Negative	Reference			Reference		
Positive	0.740	0.602-0.911	.004	0.741	0.543-1.012	.059
**HER2 status**						
Negative				Reference		
Positive	1.001	0.579-1.728	.998	0.905	0.517-1.584	.727
Unknown	1.169	0.952-1.434	.135	0.999	0.798-1.251	.994
**Surgery**						
None	Reference			Reference		
Received	0.208	0.151-0.286	<.001	0.352	0.249-0.499	<.001
**Radiotherapy**						
None	Reference			Reference		
Received	0.423	0.350-0.511	<.001	0.514	0.421-0.627	<.001
**Chemotherapy**						
None	Reference			Reference		
Received	0.663	0.527-0.835	<.001	0.604	0.461-0.793	<.001

Abbreviations: CI, Confidence Interval; ER, estrogen receptor; HR, Hazard Ratio; LN, lymph nodes; PR, progesterone receptor.

a Unmarried includes divorced, separated, single (never married), and widowed.

b Other includes American Indian/Alaskan native, or Asian/Pacific Islander.

**Table S3** presents the results of the univariate analysis, which identified several predictors of BCSS: Older age, married status, lower tumor grade, right-sided, smaller tumor size, negative LN, ER-positive, PR-positive, surgery, radiotherapy, and non-chemotherapy were all associated with improved BCSS. The multivariate analysis showed that the following were independent prognostic factors for BCSS (**Table S3**). In terms of basic information of the patients, marital status (married, HR = 0.597, 95% CI 0.399-0.893, *P *= .012) was independently related to BCSS. In terms of tumor-related information, tumor grade (grade III/IV: HR = 1.859, 95% CI 1.171-2.952, *P *= .009), laterality (right: HR = 0.636, 95% CI 0.434-0.932, *P *= .020), tumor size (Tumor size > 2.0 cm: HR = 1.744, 95% CI 1.066-2.853, *P *= .027), LN status (positive: HR = 3.907, 95% CI 2.583-5.910, *P *< .001), and PR status (positive: HR = 0.536, 95% CI 0.311-0.924, *P *= .025) were independent associated with BCSS. In terms of treatment information, surgery (received: HR = 0.141, 95% CI 0.075-0.266, *P *< .001) and radiotherapy (received: HR = 0.582, 95% CI 0.385-0.880, *P *= .010) were independently associated with BCSS in patients with IPC.

## Discussion

In this investigation, based on a large clinical cohort from the SEER database, we discovered that patients with node-negative IPC with tumors larger than 2 cm could benefit from chemotherapy. The median age of enrolled patients was 66 years old, which was older than the average diagnosis age of breast cancer. In accordance with previous study,^5^ patients over 50 years old accounted for 87.8% of this study cohort, which was also higher than the average age at the diagnosis for invasive ductal breast cancer.^13^ It was found in our study that most IPCs presented as HoR+/HER2− subtype, better histologic grade, and negative LN status (Table 1), which were also consistent with previous studies.^5^^,^^14^ Although IPC showed those seemingly favorable clinicopathological characteristics, its prognosis was not as favorable as people once thought.^5^^,^^11^ Based on the results in Table 2, determination of the appropriate therapeutic regimen for IPC still depended on risk factors, especially tumor size and lymph node involvement.

The OS of IPC patients in the chemotherapy group were significantly better than that in the non-chemotherapy group, even after balancing baseline clinicopathological factors by PSM (Table 1, Figure 3). However, this result is not consistent with some previous studies. Waks et al. reported that only a small number of patients with HR-positive breast cancer benefitted from receiving chemotherapy.^15^ In the 2015 St Gallen consensus,^16^ luminal A-like breast cancer was characterized by high ER and PR expression, negative HER2 status, and low Ki-67 index levels. Huppert et al. held the view that luminal A breast cancer patients rarely benefitted from chemotherapy, even in high-risk premenopausal populations^17^^,^^18^. However, it is worth noting that the study utilized a cyclophosphamide-based chemotherapy regimen, which differs from the taxane- or anthracycline-based regimens commonly adopted in current practice. More importantly, these studies were not conducted specifically for IPC. These inconsistent results may be mainly attributed to the unique characteristics of IPC. Although IPC is defined as invasive cancer in pathology, it was considered to be less invasive than invasive ductal carcinoma.^19^ The characterization of IPC as having relatively less aggressive biological behavior in previous studies has led to a tendency toward undertreatment in clinical practice. However, there is no evidence that a histological diagnosis of IPC warrants exemption from chemotherapy.

In the existing NCCN guidelines,^20^ there is no specific recommendation for the treatment of IPC, and the current treatment of IPC refers to that of IDC. Although most IPC presented as HoR+/HER2− subtype, lower histologic grade, and negative LN status,^5^^,^^14^ consistent with the results in Table 1, its prognosis was not more favorable than that of IDC,^11^ indicating its unique intrinsic biological behavior. It is therefore worth noting that this type of breast cancer is different from IDC, and the uniqueness of IPC should be taken into consideration in clinical practice. It is therefore worth noting that this type of breast cancer is different from IDC, and the uniqueness of IPC should be taken into consideration in clinical practice. However, few clinical trials were designed to specifically evaluate the effect of adjuvant chemotherapy on the prognosis of patients with IPC. Currently, studies specifically exploring the indications for chemotherapy in IPC are lacking.^21^ Therefore, this article provided important data reference for the prognostic impact of chemotherapy on the patients with early-stage IPC patients. It was found in this study that whether IPC patients benefit from adjuvant chemotherapy depended on LN status and tumor size, as well as tumor grade.

Intriguingly, among IPC patients with negative LNs and tumors equal to or larger than 2.0 cm in size, chemotherapy significantly improved OS in this study. However, these IPCs with negative LNs and small tumor size often fall into the category of low-risk breast cancer. According to NCCN guideline,^20^ there is a consensus on the use of genetic testing in low-risk breast cancer. Genetic testing plays an important role in identifying the individual risk of recurrence for patients with HoR+ breast cancer to avoid the side effects of unnecessary chemotherapy,^22^^,^^23^ especially for LN-negative disease. Prior to this study, we would recommend completing genetic testing for patients with luminal IPC who have node-negative tumors. However, according to the results of this study, even without genetic testing, we can still estimate that patients with node-negative IPC measuring equal to or larger than 2.0 cm can benefit significantly from chemotherapy. For patients who do not have the resources or are unwilling to complete genetic testing, this study is important supplementary evidence to guide clinical practice. In the group of patients with positive LNs and tumors equal to or larger than 2.0 cm, the number of matched cases was limited. The non-significant result was likely due to poor statistical power in certain subgroups to detect a clinically meaningful treatment effect. As documented, node-positive breast cancer has a higher risk of recurrence than node-negative breast cancer and is more likely to benefit from chemotherapy.^15^ In clinical practice, it is generally accepted that chemotherapy is usually given to patients with lymph node-positive breast cancer.^20^ Therefore, this study focused on the lymph node-negative population.

As mentioned in the 8th edition of the American Joint Committee on Cancer (AJCC) Cancer Staging Manual, the most important factor in the prognostic staging of breast cancer includes histological grades.^24^ It can be seen that accurate histological grading plays a very important guidance for the prognostic assessment of breast cancer patients. Therefore, we also performed subgroup analysis based on histologic grade. In patients with grade III/IV tumors, OS in the chemotherapy group was significantly better than that in the non-chemotherapy group; however, chemotherapy showed no beneficial effect on grade I/II tumors. The above results indicate that chemotherapy might be more valuable for patients with high histologic grade IPC.

There are few studies devoted to IPC therapy due to the rarity of the disease, but the application of new biotechnology might help to pioneer a more suitable treatment for this rare tumor. For example, Li et al. carried out a drug sensitivity test by constructing organoids to explore the curative effect of endocrine drugs on IPC patients.^25^ Using immunohistochemical markers to evaluate myoepithelium may help better indicate the malignancy of IPC and guide the treatment of IPC in the future.^26^ However, the research on these methods is still in progress. Our research findings can provide important guidance for the decision-making of current clinical treatment options.

### Study limitations

Considering that this study covered a large number of cases, had a long follow-up period, and employed the PSM approach to balance confounding factors, it retains high credibility. However, our study still has some limitations. Compared to traditional multivariable regression, PSM offers an intuitive framework to mimic randomization and visualize group balance, which is particularly useful when treatment assignment is not random. However, PSM only accounts for observed covariates and does not control for unmeasured confounding. That is to say, the retrospective nature of this study might inevitably introduce selection bias, even with the use of PSM to balance baseline factors. Second, chemotherapy regimens were not available from the SEER database, which may affect the analysis of chemotherapy effects; however, this is a common limitation of all studies based on SEER and can only be overcome with more comprehensive data collection. Third, records for Ki-67 expression were also not available in the SEER database, which conceals the prognostic factor from researchers.

Although our study provides evidence that patients with negative LNs and tumors equal to or larger than 2.0 cm and patients with grade III/IV tumors can benefit from chemotherapy, further clinical trials or prospective studies are expected to validate our results.

## Conclusion

This study demonstrated that younger age, married status, smaller tumor size, negative LN status, and receipt of surgery, radiotherapy, and chemotherapy were associated with improved OS in patients with IPC. After PSM, a significant improvement in OS was observed among patients who received chemotherapy. We suggest that patients with negative LNs should be considered for chemotherapy if their tumors are ≥ 2.0 cm in size. In addition, patients with grade III or IV IPC may also benefit from chemotherapy. This study provides important evidence to support chemotherapy decision-making in patients with IPC.

## Supplementary Material

oyaf422_Supplementary_Data

## Data Availability

The datasets analyzed for this study can be found in the SEER database (http://www. seer. cancer. gov/seerstat). Further inquiries can be directed to the corresponding author.
