# Supplementary material for: Chemotherapy improves survival for patients with lymph node-negative invasive papillary breast cancer with tumors ≥2 cm: a SEER population-based study
Source: Oncologist. 2025 Dec 22;31(2):oyaf422. doi: 10.1093/oncolo/oyaf422 (PMC12832947; doi:10.1093/oncolo/oyaf422)
Supplement: oyaf422_Supplementary_Data [file oyaf422_supplementary_data.zip › Supplementary information0607 clean.docx]

**Chemotherapy improves survival for patients with lymph node-negative** **invasive papillary breast cancer with tumors ≥2 cm: a SEER population-based study.**

**Supplementary Figure S1.** Comparison of the OS and the BCSS of LN-positive patients between the chemotherapy and the non-chemotherapy groups stratified by tumor size after matching. A, OS in the T ＜2.0 cm group; B, BCSS in the T ＜2.0 cm group; C, OS in the T ≥2.0 cm group; D, BCSS in the T ≥2.0 cm group. OS, overall survival; BCSS, breast cancer-specific survival; T, tumor size; LN, lymph node.

**Alt Text:** Kaplan-Meier survival curves comparing overall survival and breast cancer-specific survival for lymph node-positive patients between chemotherapy and non-chemotherapy groups, stratified by tumor size. Panels A and B show overall survival and breast cancer-specific survival for the T < 2.0 cm group, while panels C and D show overall survival and breast cancer-specific survival for the T ≥ 2.0 cm group. The x-axis represents survival months, and the y-axis represents survival probability.

**Supplementary Figure S2.** Comparison of the OS and the BCSS of patients between the chemotherapy and the non-chemotherapy groups stratified by grade after matching. A, OS in the grade I/II group; B, BCSS in the grade I/II group; C, OS in the grade III/IV group; D, BCSS in the grade III/IV group. OS, overall survival; BCSS, breast cancer specific-survival.

**Alt Text:** Kaplan-Meier survival curves comparing overall survival and breast cancer-specific survival for patients between chemotherapy and non-chemotherapy groups, stratified by tumor grade. Panels A and B show overall survival and breast cancer-specific survival for the grade I/II group, while panels C and D show overall survival and breast cancer-specific survival for the grade III/IV group. The x-axis represents survival months, and the y-axis represents survival probability.

| **Table S1 5-year overall survival (OS) and breast cancer-specific survival (BCSS) of the two groups.** | | | | | |
| --- | --- | --- | --- | --- | --- |
| Groups | 5-year OS | |  | 5-year BCSS | |
|  | Before matching | After matching |  | Before matching | After matching |
| **Chemotherapy** | 89.0% | 91.3% | | 91.6% | 94.0% |
| **Non-chemotherapy** | 85.1% | 83.4% | | 97.5% | 96.6% |
| ***p*-value** | <0.001 | 0.0123 | | 0.001 | 0.0987 |
| **Total** | 86.00% | 87.30% | | 96.10% | 95.30% |

| **Table S2 10-year overall survival (OS) and breast cancer-specific survival (BCSS) of the two groups.** | | | | | |
| --- | --- | --- | --- | --- | --- |
| Groups | 10-year OS | |  | 10-year BCSS | |
|  | Before matching | After matching |  | Before matching | After matching |
| **Chemotherapy** | 75.8% | 76.6% | | 84.1% | 87.4% |
| **Non-chemotherapy** | 68.5% | 68.5% | | 94.8% | 93.1% |
| ***p*-value** | <0.001 | 0.0123 | | 0.001 | 0.0987 |
| **Total** | 70.1% | 72.5% | | 92.2% | 90.2% |

| **Table S3. Univariate and multivariate Cox regression model analysis of BCSS between the chemotherapy group and the non-chemotherapy group.** | | | | | | | | |
| --- | --- | --- | --- | --- | --- | --- | --- | --- |
| Characteristics | Univariate analysis | | |  | | Multivariate analysis | | |
|  | HR | 95% CI | *p*-value | | HR | | 95% CI | *p*-value |
| **Age** |  |  |  | |  | |  |  |
| <50 | Reference |  |  | | Reference | |  |  |
| ≥50 | 0.622 | 0.403-0.959 | 0.032 | | 1.090 | | 0.687-1.729 | 0.716 |
| **Marital Status** |  |  |  | |  | |  |  |
| Unmarried^a^ | Reference |  |  | | Reference | |  |  |
| Married | 0.548 | 0.373-0.807 | 0.002 | | 0.597 | | 0.399-0.893 | 0.012 |
| Unknown | 1.233 | 0.613-2.477 | 0.557 | | 1.210 | | 0.590-2.479 | 0.603 |
| **Race** |  |  |  | |  | |  |  |
| White | Reference |  |  | | Reference | |  |  |
| Black | 1.375 | 0.890-2.125 | 0.151 | | 1.085 | | 0.684-1.721 | 0.730 |
| Other^b^ | 0.537 | 0.260-1.109 | 0.093 | | 0.556 | | 0.248-1.078 | 0.078 |
| **Grade** |  |  |  | |  | |  |  |
| I/II | Reference |  |  | | Reference | |  |  |
| III/IV | 3.529 | 2.397-5.197 | <0.001 | | 1.859 | | 1.171-2.952 | 0.009 |
| Unknown | 1.230 | 0.680-2.228 | 0.493 | | 0.869 | | 0.468-1.614 | 0.657 |
| **Laterality** |  |  |  | |  | |  |  |
| Left | Reference |  |  | | Reference | |  |  |
| right | 0.665 | 0.457-0.967 | 0.033 | | 0.636 | | 0.434-0.932 | 0.020 |
| **Tumor Size** |  |  |  | |  | |  |  |
| ≤2.0 | Reference |  |  | | Reference | |  |  |
| >2.0 | 3.918 | 2.466, 6.226 | <0.001 | | 1.744 | | 1.066-2.853 | 0.027 |
| Unknow | 2.954 | 1.790, 4.873 | <0.001 | | 2.027 | | 1.165-3.527 | 0.012 |
| **LN Status** |  |  |  | |  | |  |  |
| Negative | Reference |  |  | | Reference | |  |  |
| Positive | 4.722 | 3.286-6.785 | <0.001 | | 3.907 | | 2.583-5.910 | <0.001 |
| **ER Status** |  |  |  | |  | |  |  |
| Negative | Reference |  |  | | Reference | |  |  |
| Positive | 0.381 | 0.254-0.573 | <0.001 | | 0.930 | | 0.506-1.708 | 0.814 |
| **PR Status** |  |  |  | |  | |  |  |
| Negative | Reference |  |  | | Reference | |  |  |
| Positive | 0.348 | 0.240-0.505 | <0.001 | | 0.536 | | 0.311-0.924 | 0.025 |
| **HER2 Status** |  |  |  | |  | |  |  |
| Negative | Reference |  |  | | Reference | |  |  |
| Positive | 1.448 | 0.571-3.674 | 0.436 | | 0.760 | | 0.293-1.971 | 0.572 |
| Unknown | 1.226 | 0.810-1.855 | 0.335 | | 0.814 | | 0.3509-1.303 | 0.392 |
| **Surgery** |  |  |  | |  | |  |  |
| None | Reference |  |  | | Reference | |  |  |
| Received | 0.155 | 0.088-0.273 | <0.001 | | 0.141 | | 0.075-0.266 | <0.001 |
| **Radiotherapy** |  |  |  | |  | |  |  |
| None | Reference |  |  | | Reference | |  |  |
| Received | 0.528 | 0.362-0.770 | <0.001 | | 0.582 | | 0.385-0.880 | 0.010 |
| **Chemotherapy** |  |  |  | |  | |  |  |
| None | Reference |  |  | | Reference | |  |  |
| Received | 3.003 | 2.088-4.318 | <0.001 | | 1.435 | | 0.915-2.249 | 0.116 |
| Abbreviations: CI, Confidence Interval; ER, estrogen receptor; HR, Hazard Ratio; LN, lymph nodes; PR, progesterone receptor.  ^a^Unmarried includes divorced, separated, single (never married), and widowed.  ^b^Other includes American Indian/Alaskan native, or Asian/Pacific Islander. | | | | | | | | |
