# Supplementary figures and images for: Chemotherapy improves survival for patients with lymph node-negative invasive papillary breast cancer with tumors ≥2 cm: a SEER population-based study
Source: Oncologist. 2025 Dec 22;31(2):oyaf422. doi: 10.1093/oncolo/oyaf422 (PMC12832947; doi:10.1093/oncolo/oyaf422)

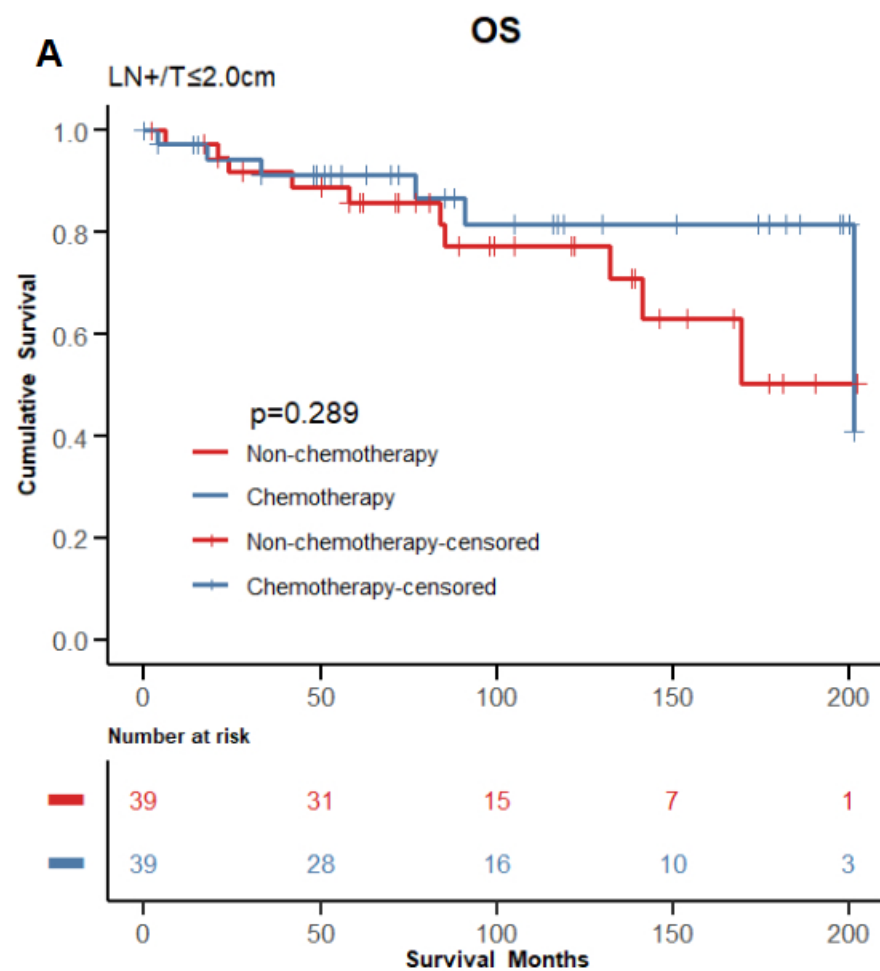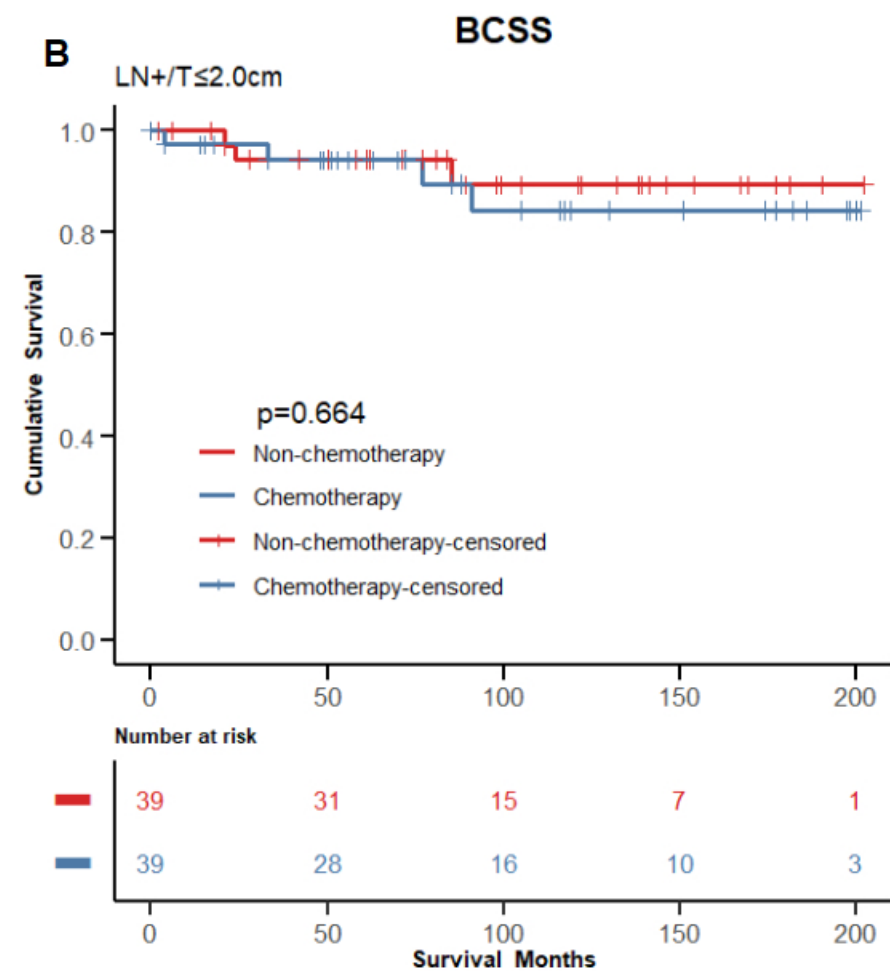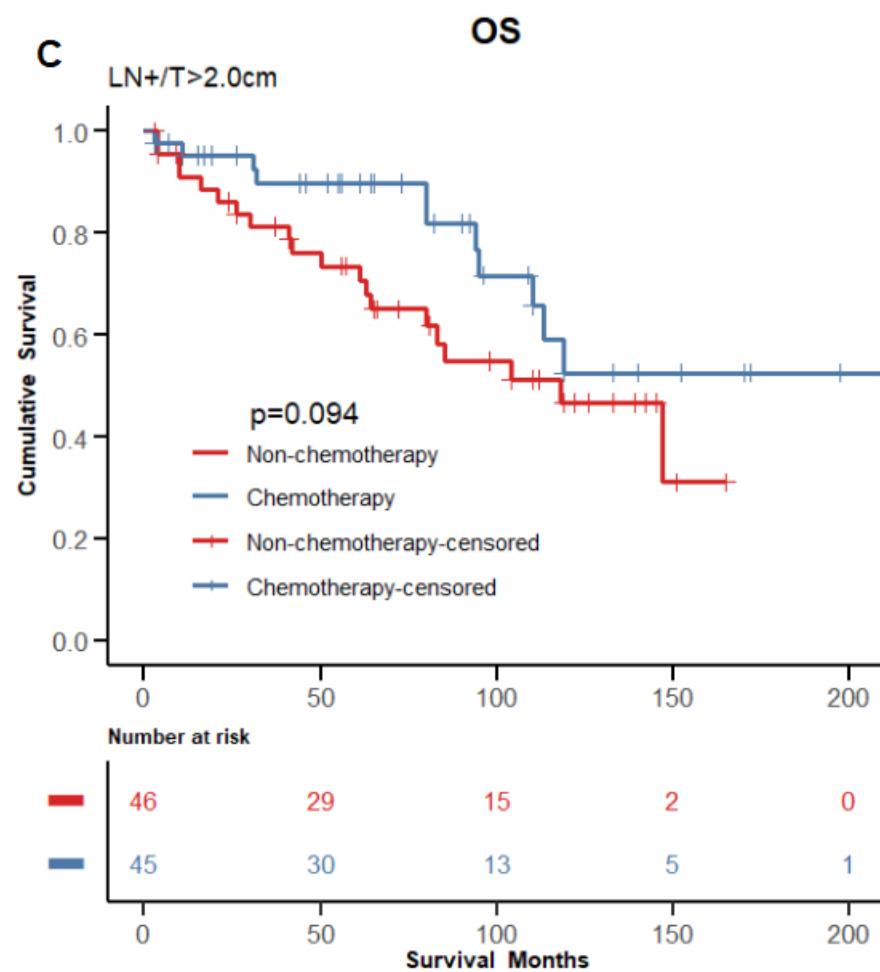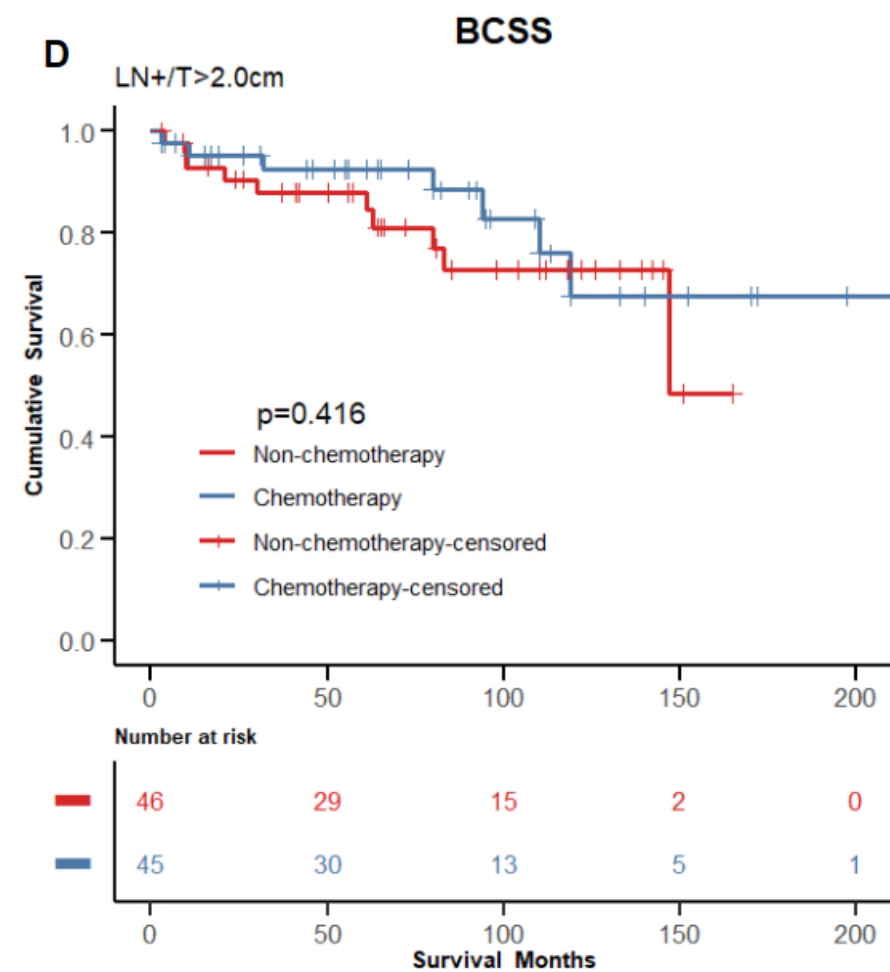

Supplement: oyaf422_Supplementary_Data [file oyaf422_supplementary_data.zip › Supplementary Figure S1.pdf]

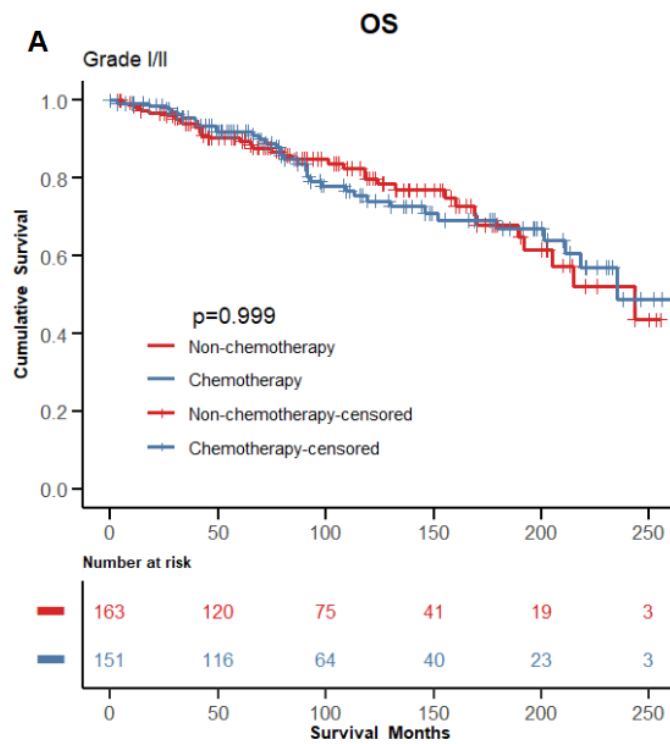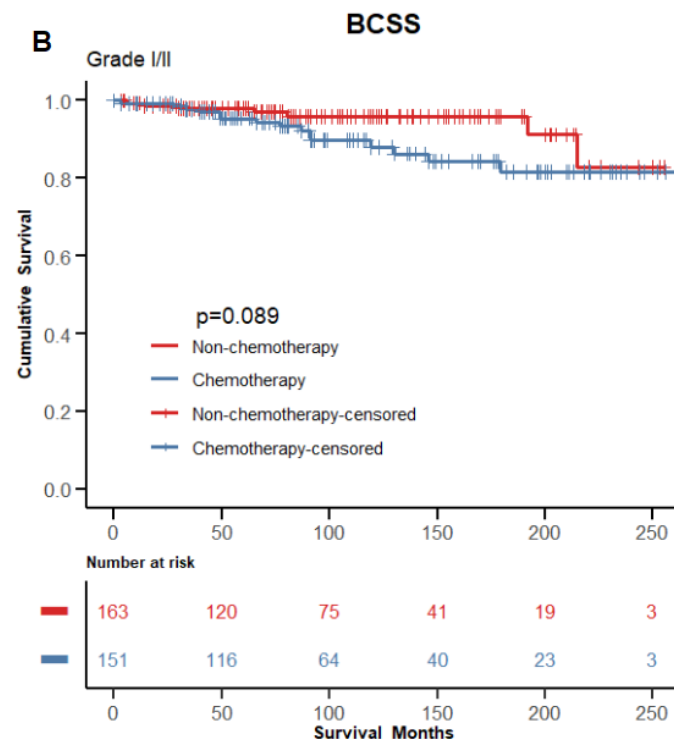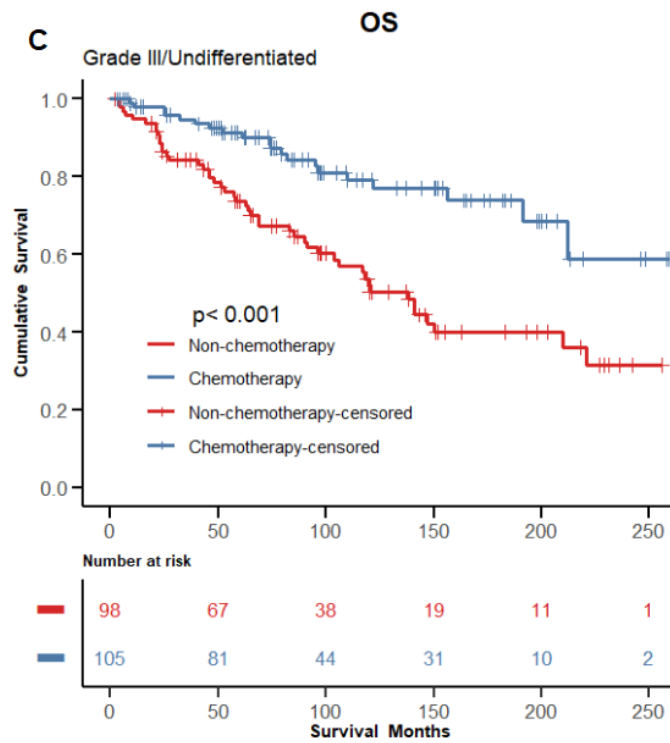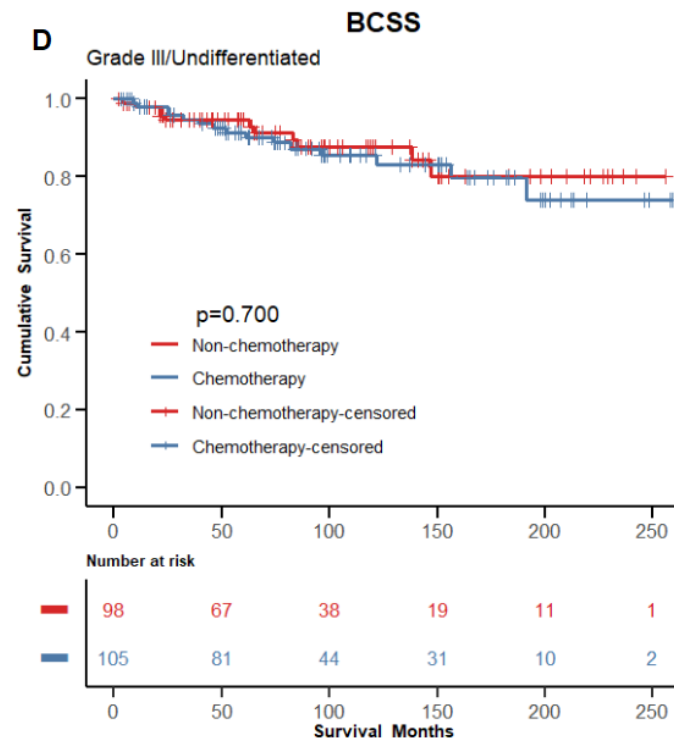

Supplement: oyaf422_Supplementary_Data [file oyaf422_supplementary_data.zip › Supplementary Figure S2.pdf]
